# Luciferase expression and bioluminescence does not affect tumor cell growth *in vitro *or *in vivo*

**DOI:** 10.1186/1476-4598-9-299

**Published:** 2010-11-22

**Authors:** Jessamy C Tiffen, Charles G Bailey, Cynthia Ng, John EJ Rasko, Jeff Holst

**Affiliations:** 1 Gene and Stem Cell Therapy Program, Centenary Institute, University of Sydney, Camperdown NSW 2050, Australia; 2 Cell and Molecular Therapies, Royal Prince Alfred Hospital, Camperdown NSW 2050, Australia

## Abstract

Live animal imaging is becoming an increasingly common technique for accurate and quantitative assessment of tumor burden over time. Bioluminescence imaging systems rely on a bioluminescent signal from tumor cells, typically generated from expression of the firefly luciferase gene. However, previous reports have suggested that either a high level of luciferase or the resultant light reaction produced upon addition of D-luciferin substrate can have a negative influence on tumor cell growth. To address this issue, we designed an expression vector that allows simultaneous fluorescence and luminescence imaging. Using fluorescence activated cell sorting (FACS), we generated clonal cell populations from a human breast cancer (MCF-7) and a mouse melanoma (B16-F10) cell line that stably expressed different levels of luciferase. We then compared the growth capabilities of these clones *in vitro *by MTT proliferation assay and *in vivo *by bioluminescence imaging of tumor growth in live mice. Surprisingly, we found that neither the amount of luciferase nor biophotonic activity was sufficient to inhibit tumor cell growth, *in vitro *or *in vivo*. These results suggest that luciferase toxicity is not a necessary consideration when designing bioluminescence experiments, and therefore our approach can be used to rapidly generate high levels of luciferase expression for sensitive imaging experiments.

## Findings

Bioluminescence imaging (BLI) is an increasingly popular technique for quantitatively assessing tumor growth and the effects of therapy over time [1]. The sensitivity and accuracy of *in vivo *BLI systems offers several advantages over traditional methods of measuring subcutaneous tumors using calipers [2-9]. Typically cancer cells are engineered to express the firefly luciferase gene and are engrafted into mice to form tumors [10]. Following an intraperitoneal injection of D-luciferin, the luciferase enzyme will catalyze this substrate into oxyluciferin, requiring the presence of oxygen, and cofactors such as adenosine triphosphate (ATP) and Mg^2+ ^ions [11]. The resulting light photons generated by this reaction are captured non-invasively with a charge-coupled device (CCD) camera mounted within the BLI system [12]. Successful BLI requires prior modification of the cancer cell line with the luciferase gene, however little is known about the effect this may have on normal cell function [13]. To date, the only evidence of a detrimental effect of biophotonic emissions on cell function was in a luciferase-expressing ovarian cancer cell line that showed a high level of luciferase reduced tumor growth *in vivo *[14]. It was suggested that build up of oxyluciferin during repeated BLI might cause oxidative damage to the cells. Limiting cofactors in the luciferase-luciferin reaction include oxygen and ATP [15]; therefore high levels of biophotonic activity may place extra demand for energy on the cells, possibly leading to growth inhibition. One report even suggests the use of luciferase in photodynamic therapy following a 90% reduction in the survival of NIH3T3 mouse fibroblasts, which were stably expressing luciferase and incubated with a photosensitizer [16]. However, doubts remain as to whether luciferase can generate enough photons to significantly inhibit the growth of cancer cells.

**Figure 1:**
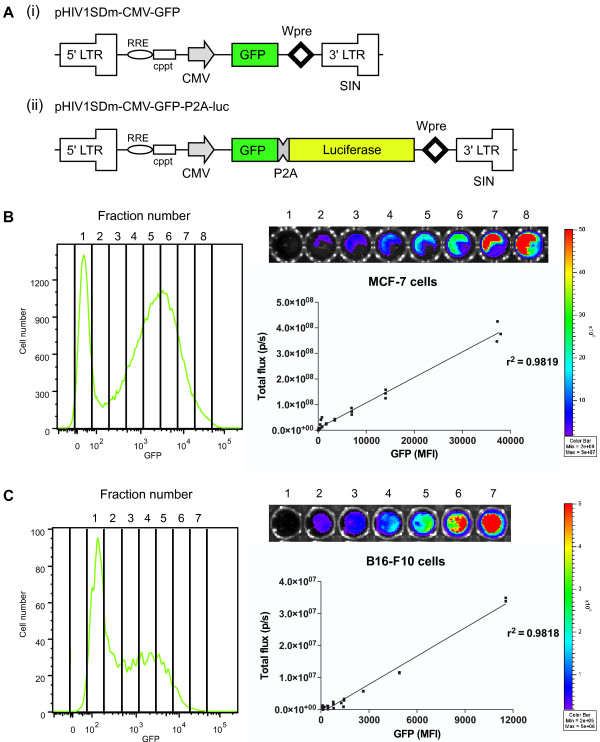
**Lentiviral vectors designed to achieve equimolar expression of GFP and luciferase**. (A) The third-generation lentiviral expression vector pHIV1SDm [17] containing 5' and 3' LTRs, long terminal repeat; RRE, rev response element; cPPT, central polypurine tract; CMV, cytomegalovirus promoter; GFP, enhanced green fluorescent protein; P2A, porcine teschovirus-1 2A motif; Wpre, woodchuck hepatitis post-transcriptional regulatory element; and self-inactivating (SIN) 3'LTR. The vectors contained (i) GFP alone as a control, or (ii) a GFP and firefly luciferase cassette (GFP-P2A-luc). (B) MCF-7 or (C) B16-F10 cells were transduced with a lentiviral vector containing the GFP-P2A-luc cassette and purified into 7 or 8 different populations based on increasing GFP expression by FACS. B16-F10 (20, 000) or MCF-7 (10, 000) cells/well were deposited in a black 96-well plate and luciferase expression was immediately confirmed upon addition of D-luciferin substrate (150 μg/mL final concentration). Luciferase bioluminescence was quantified using the Xenogen IVIS-100 and Living Image software (Caliper Life Sciences). GFP mean fluorescence intensity (MFI) vs. luciferase total flux (p/s = photons/second) was plotted and linear regression calculated for the line of best fit (n = 3). All statistical analyses were performed using GraphPad Prism 5.01.

To address the issue of potential luciferase toxicity resulting from BLI, we designed a lentiviral vector that enabled reliable selection of the level of luciferase expression in cells. This lentiviral vector [17] encodes green fluorescent protein (GFP) alone (Figure 1Ai) or GFP linked to firefly luciferase (Figure 1Aii) by a 23 amino acid picornaviral 2A-like sequence from the porcine teschovirus-1 (P2A) [18]. This GFP-P2A-luc cassette permits equimolar expression of GFP and luciferase via a ribosomal skipping mechanism. Human MCF-7 breast cancer or mouse B16-F10 melanoma cell lines were stably transduced and then purified by FACS into different fractions based on increasing levels of GFP expression. Cell fractions were then subjected to BLI to demonstrate that the level of GFP directly correlated to the level of luciferase expression in MCF-7 cells (r^2 ^= 0.9819; Figure 1B) and B16-F10 cells (r^2 ^= 0.9818; Figure 1C). MCF-7 cells were substantially brighter than B16-F10 cells, both in luminescence and GFP expression (Figure 1B, C). This may result from differences in transduction efficiencies, promoter efficiency, or the known absorbance of photons by melanin in the B16-F10 cells [19].

**Figure 2:**
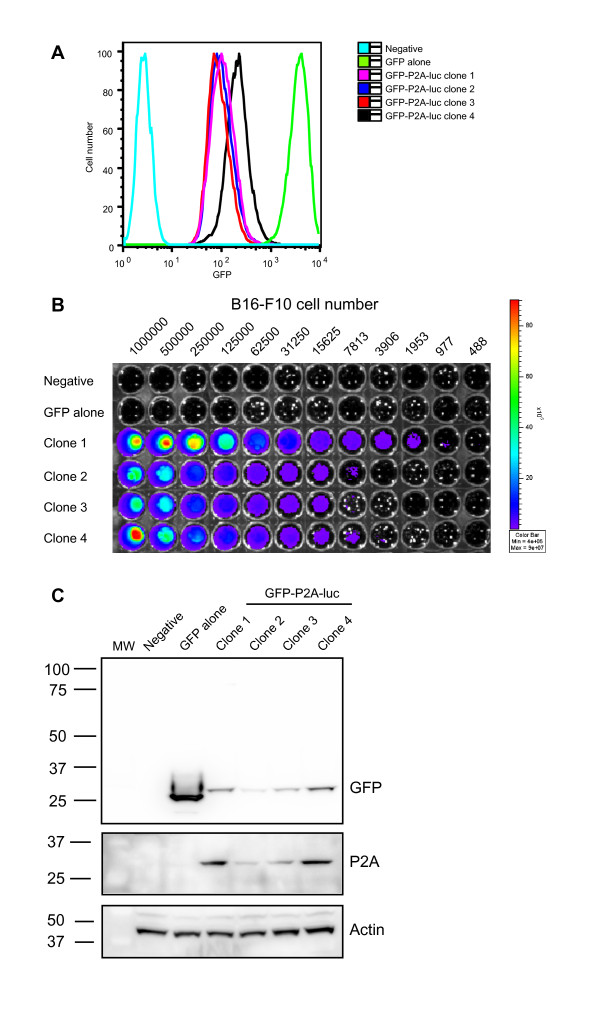
**Isolation of clones that stably express GFP and luciferase**. B16-F10 cells were transduced with the GFP-P2A-luc lentiviral vector and GFP- positive cells were purified by FACS (> 95%). (A) Single cell clones were isolated by limiting dilution, expanded *in vitro *and tested to confirm stable expression of GFP. The specificity and sensitivity of luciferase expression was demonstrated by serial dilution of the B16-F10 clones and appropriate controls (negative = untransduced, or cells transduced with GFP only). BLI images were taken prior to or following the addition of D-luciferin substrate (B) using a Xenogen IVIS-100. (C) Complete separation of GFP and luciferase protein was confirmed by SDS-PAGE and immunoblotting using anti-GFP (Cat. # 632380, BD Bioscience) and anti-P2A antibodies [18]. Separated GFP protein migrated at 27 kDa, whereas the GFP-P2A product will migrate at a higher molecular weight (~29 kDa). Un-separated GFP-P2A-luc protein was predicted to migrate at 90 kDa and was not present. Actin was used as a loading control and detected also by immunoblotting (Cat. # A2103, Sigma).

Clonal populations of MCF-7 and B16-F10 cells expressing a homogenous level of luciferase, were generated by expansion of individual FACS-purified cells. Several different clones were isolated from each cell type to represent different levels of luciferase expression. Following expansion, each clone was analyzed by flow cytometry to ensure GFP expression remained stable after several weeks in culture (Figure 2A). To demonstrate the stability of the biophotonic reaction and the sensitivity of detection, B16-F10 clones were serially diluted in culture and subjected to BLI following addition of D-luciferin (Figure 2B). The lack of photons emitted by untreated cells or cells transduced with GFP-containing vector alone indicated the specificity of the bioluminescence reaction. To assess how luciferase bioluminescence in our clones compared to levels reported by Brutkiewicz *et al*, we used a combination of luminometry and BLI (Additional File 1). The biophotonic emission of the brightest clone was 226, 818 counts per second (CPS)/μg of protein, that represents a 1222-fold increase in luciferase bioluminescence compared to negative cells. We believe such levels are comparable to, or greater, than those previously reported [14]. Heterologous protein levels were also assessed by SDS-PAGE and immunoblotting using antibodies against GFP or the P2A sequence and indicate a range of expression between clones (Figure 2C). The P2A epitope (~2 kDa) remains attached to GFP post-separation, resulting in the higher molecular weight bands observed in cells expressing the GFP-P2A-luc cassette, compared to GFP alone at 27 kDa. Complete separation of GFP from luciferase had occurred as indicated by the absence of a fusion protein at 90 kDa (Figure 2C).

**Figure 3:**
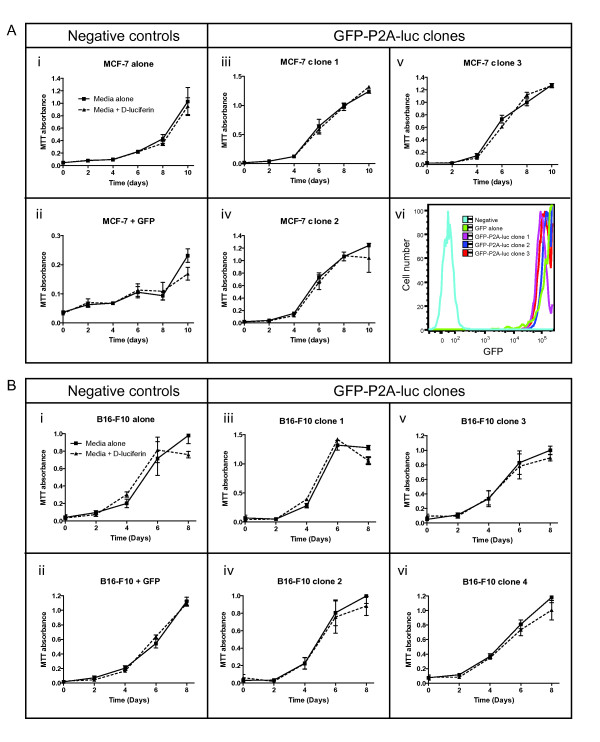
**The luciferase gene or luciferase bioluminescence activity does not affect the growth of cancer cell lines *in vitro***. (A) MCF-7 cells were assessed for proliferation by MTT assay: i) untransduced; ii) transduced with GFP alone; iii-v) transduced with GFP-P2A-luc (clones 1-3); vi) representative flow cytometric analysis of GFP expression in cells i-v. (B) B16-F10 cells were assessed for proliferation by MTT assay: i) untransduced; ii) transduced with GFP alone; iii-vi) transduced with GFP-P2A-luc (clones 1-4). Cells were treated with D-luciferin substrate (150 μg/mL final concentration) diluted in media or media alone at 2-day intervals. Following 30 min incubation at 37°C in the dark, cells were washed once with PBS and the normal growth medium was replaced. Proliferation was measured by addition of MTT substrate overnight, followed by measuring the absorbance at 572 nm. Data is shown as the mean ± s.e.m. from 3 independent experiments.

To investigate whether biophotonic activity or the luciferase gene itself had a negative influence on cell growth *in vitro*, proliferation assays were performed. MCF-7 (Figure 3A) or B16-F10 (Figure 3B) clones expressing different levels of GFP-P2A-luc plus negative controls were periodically treated with D-luciferin and assessed for proliferation by MTT assay. All cell types displayed similar growth dynamics, indicating neither the presence of the luciferase gene, nor biophotonic activity was sufficient to inhibit growth compared to controls (Figure 3A, B). This occurred despite the high expression of GFP-P2A-luc observed in the MCF-7 cells by flow cytometry (Figure 3Avi). The viability of luciferase-expressing clones was also assessed using a live/dead fluorescent stain in conjunction with flow cytometry analysis (Additional File 2). The results revealed no significant difference in cell viability between clones expressing different levels of luciferase or between cells periodically treated with D-luciferin. To determine whether these observations extended to non-clonal populations of cells expressing a wider range of luciferase levels, cell growth was assessed in multiple cell types (Additional File 3). Cells transduced with GFP-P2A-luc were purified by FACS into mixed populations expressing no, low, medium or high levels of luciferase, before being cultured with or without periodic D-luciferin treatments. MTT assays indicated that neither the level of luciferase, nor biophotonic activity, affected tumor cell growth in MCF-7, B16-F10, ACHN renal and CT26 colon carcinoma cells (Additional File 3B-E respectively). Furthermore, there was no difference between FACS-purified populations of GFP- and GFP-P2A-luc-expressing B16-F10 cells with similar GFP expression (Additional File 3F). These observations are supported by a previous report that found luciferase bioluminescence was not sufficient to generate photodynamic toxicity *in vitro *in a range of cell lines, even in the presence of photosensitizers [20].

**Figure 4:**
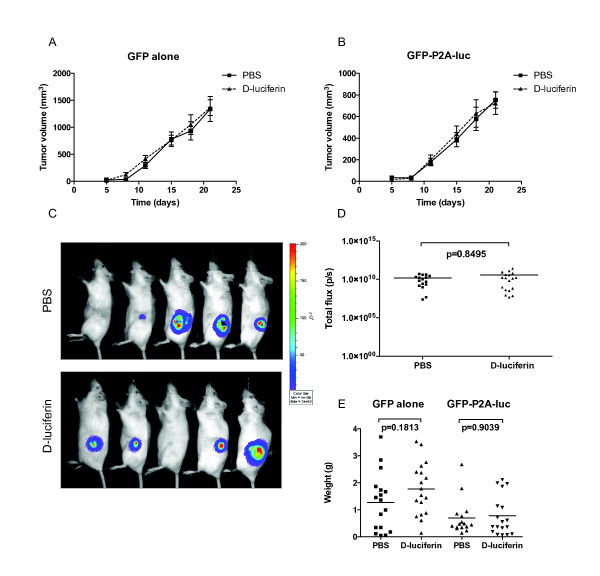
**The luciferase gene or luciferase bioluminescence does not affect the growth of tumors in mice**. B16-F10 cells (1 × 10^6^) expressing GFP alone or GFP-P2A-luc (clone 4) were injected subcutaneously into the right or left flank respectively of white C57Bl/6 mice. Mice received bi-weekly injections of either PBS or D-luciferin (150 mg/kg) and tumor growth was recorded using calipers for GFP alone (A) or GFP-P2A-luc (B). Ellipsoid volume was determined using the formula π/6 × (L × W)^3/2 ^[21]. After 21 days, bioluminescent imaging was performed on tumors expressing GFP-P2A-luc (C) using the Xenogen IVIS-100. (D) Images were analyzed using Living Image software and represented as total flux measurements in photons/second. (E) Weight data was recorded from the excised tumors at sacrifice day 21. Statistical significance was assessed using non-parametric (Mann-Whitney) analysis using GraphPad Prism 5.01. Data is the mean ± s.e.m. from 4 independent experiments.

To validate these findings *in vivo*, we performed BLI in a subcutaneous mouse tumor model. B16-F10 clonal cells expressing GFP alone or GFP-P2A-luc (clone 4) were injected subcutaneously into the right or left hand flanks respectively of white C57Bl/6 mice. Animals were divided into two cohorts; one received bi-weekly intraperitoneal injections of phosphate buffered saline (PBS), and the other D-luciferin. Tumor growth was monitored using caliper measurements (Figure 4A, B) and on the final day tumors expressing GFP-P2A-luc from both groups were subjected to BLI (Figure 4C, D). Following sacrifice, tumors were extracted and weighed for comparison (Figure 4E). Consistent with our *in vitro *findings, neither the presence of the luciferase gene, nor biophotonic activity significantly inhibited tumor growth *in vivo*. This finding was validated not only by caliper measurements but also by endpoint BLI and tumor weight data. No inhibitory effect was observed despite the high level of GFP-P2A-luc expression of B16-F10 clone 4. Caliper and weight data did reveal an overall size difference between tumors expressing GFP alone and GFP-P2A-luc (Figure 4A, B and 4E). However, it is impossible to determine whether this difference was due to the presence of the luciferase gene, because these tumors arose from separate single cell clones with differing growth characteristics. Immunohistochemical analysis of Ki67 and cleaved caspase 3 using paraffin-embedded sections from these tumors revealed no significant difference in proliferation or apoptosis respectively (Additional File 4). This occurred regardless of luciferase gene expression or D-luciferin treatment. Taken together, these data suggest that the differences between the GFP- and GFP-P2A-luc-expressing clones are most likely due to interclonal variation rather than a direct affect of the luciferase gene.

Future work is needed to investigate the possibility of an immune response against tumor cells expressing firefly luciferase or P2A; however as most researchers perform xenograft experiments in immune-compromised animals, the immunogenicity of luciferase is unlikely to be a significant concern. The use of clonal cell populations is useful to ensure a homogenous expression of luciferase, but problems may arise due to interclonal variation (Figure 4A, B). Based on the stoichiometric expression of GFP and luciferase protein, our approach also allows selection of a polyclonal population of cells using a narrow band of GFP expression (Figure 1B, C), thus minimizing any interclonal variation. It has been suggested that a hypoxic tumor environment can lead to a reduction in intracellular ATP levels, that in turn may result in an underestimation of BLI [15]. Our vector provides a solution to this problem in that imaging can be performed using luminescence or fluorescence, to ensure comparable measurements.

Our vector represents a versatile tool for BLI in that fluorescence from GFP-positive cells correlates directly with luciferase expression levels. Contrary to previous reports however [14,16], we found that neither a high level of luciferase expression, nor biophotonic activity had a detrimental effect on cancer cell growth *in vitro *or *in vivo*. In light of these data, we conclude that oxyluciferin toxicity is not an important consideration when designing BLI experiments.

## List of abbreviations

FACS: Fluorescence activated cell sorting. MTT: 3-(4,5-Dimethylthiazol-2-yl)-2,5-diphenyltetrazolium bromide. BLI: Bioluminescence imaging. P2A: Porcine teschovirus-1 2A sequence. SDS-PAGE: Sodium dodecyl sulfate polyacrylamide gel electrophoresis. PCR: Polymerase chain reaction.

## Competing interests

The authors declare that they have no competing interests.

## Authors' contributions

JT characterized the clonal cell populations *in vitro*, established the bioluminescent tumor model in mice and drafted the manuscript. CB provided technical expertise in viral vector construction and production, CN assisted with PCR and vector cloning. JR contributed to data interpretation and provided intellectual input. JH conceived the study, generated the cell clones and provided intellectual input. All authors read and approved the final manuscript.

## Supplementary Material

Additional file 1 **Quantifying luciferase bioluminescence**. B16-F10 and MCF-7 cells were transduced with the GFP-P2A-luc-containing lentiviral vector and GFP-positive cells were purified by FACS (> 95%). Single cell clones were isolated by limiting dilution, and expanded *in vitro*. B16-F10 and MCF-7 (2 × 10^6^) clonal cells were lyzed using 5X reporter lysis buffer from the Luciferase Assay System (Promega). The protein concentration was then determined using a protein assay (Micro BCA, Thermo Scientific). Cell lysate (5 μL) was mixed with 100 μL of Luciferase Assay Reagent in each well of a white 96-well plate using the microinjector on the Victor2 Wallac plate reader (Perkin Elmer). (A) Luminometry was used to measure light emissions in counts per second (CPS) over a 5 second period. Data is quantified in CPS/μg of total protein for each clone and represents 3 individual experiments performed in triplicate. (B) Luciferase bioluminescence was imaged using a Xenogen IVIS-100 by serial dilution of clones and appropriate controls (negative = untransduced, or cells transduced with GFP only). Bioluminescent images were taken following the addition of D-luciferin substrate. (C) Wells containing 1 × 10^6 ^cells were quantified using Living Image software and represented as total flux measurements in photons/second. Click here for file

Additional file 2 **Luciferase bioluminescence does not affect tumor cell viability *in vitro***. B16-F10 and MCF-7 clonal cells that stably express luciferase were seeded in 12-well plates (2 × 10^3^/well). Cells were treated with D-luciferin substrate diluted in media or media alone at 2-day intervals. Following 30 min incubation at 37°C in the dark, cells were washed once with PBS and the normal growth medium was replaced. Cell viability was measured after 8-10 days in culture using the LIVE/DEAD Fixable Dead Cell Stain Kit (Invitrogen). The violet fluorescent dye (1:500) was used to distinguish between live and dead cell populations using the BD FACSCanto flow cytometer and analyzed using Flowjo version 8.1 (Treestar). The live cell populations are represented for (A) B16-F10 cells and (B) MCF-7 cells. Click here for file

Additional file 3 **A range of luciferase bioluminescence does not affect tumor cell viability in multiple cell types**. (A) Cells were transduced with a lentiviral vector containing the GFP-P2A-luc cassette and purified into negative, low, medium or high GFP-expressing populations by FACS. Cells (400/well) were deposited directly into 96-well plates and were treated with D-luciferin substrate diluted in media or media alone at 2-day intervals. Following 30 min incubation at 37°C in the dark, cells were washed once with PBS and the normal growth medium was replaced. Cell viability was measured after 8-10 days in culture by addition of MTT substrate overnight, followed by measuring the absorbance at 572 nm for (B) MCF-7, (C) B16-F10 (D) ACHN and (E) CT26 cells. A mixed population of B16-F10 cells were transduced with lentivirus containing the GFP-P2A-luc cassette or GFP alone. (F) Identical populations of GFP expressing cells were purified by FACS and cell viability was assessed by addition of MTT substrate overnight, followed by measuring the absorbance at 572 nm. Data is shown as the mean ± s.e.m. from 3 independent experiments. Click here for file

Additional file 4 **Luciferase bioluminescence does not affect tumor cell proliferation or viability *in vivo***. B16-F10 tumors expressing GFP-P2A-luc or GFP alone were excised from animals and fixed in 10% neutral buffered formalin. Tumors were embedded in paraffin, cut into serial sections and mounted on lysine-coated slides. Tumor sections were stained using a Bond Max autostainer (Leica Microsystems) with an anti-Ki67 (Cat. # ORG-8772, Novocastra) or an anti-cleaved (c) caspase-3 monoclonal antibody (Cat. # 9664). A secondary antibody conjugated to alkaline phosphatase followed by chromogen staining (Fast Red) was used to distinguish between melanin and positive staining in melanocytes. Sections were counterstained with haematoxylin. (A) Ki67 staining was assessed in: i) normal tonsil tissue (positive control); ii) GFP alone tumors treated with PBS; iii) GFP alone tumors treated with D-luciferin; iv) GFP-P2A-luc tumors treated with PBS; and v) GFP-P2A-luc tumors treated with D-luciferin. Nuclear staining is indicated by arrows. (B) c-caspase-3 staining was assessed in: i) Etoposide treated Jurkat cells (positive control); ii) GFP alone tumors treated with PBS; iii) GFP alone tumors treated with D-luciferin; iv) GFP-P2A-luc tumors treated with PBS; and v) GFP-P2A-luc tumors treated with D-luciferin. Cytoplasmic staining is indicated by arrows. Slides were then scanned and whole sections were assessed for positive staining using the ACIS III system and software (Dako). Statistical significance was assessed using non-parametric (Mann-Whitney) analysis using GraphPad Prism 5.01. Data is shown as the mean ± s.e.m. from 3 tumor sections derived from one experiment. Click here for file
