# Supplementary figures and images for: Luciferase expression and bioluminescence does not affect tumor cell growth in vitro or in vivo
Source: Mol Cancer. 2010 Nov 22;9:299. doi: 10.1186/1476-4598-9-299 (PMC3002927; doi:10.1186/1476-4598-9-299)

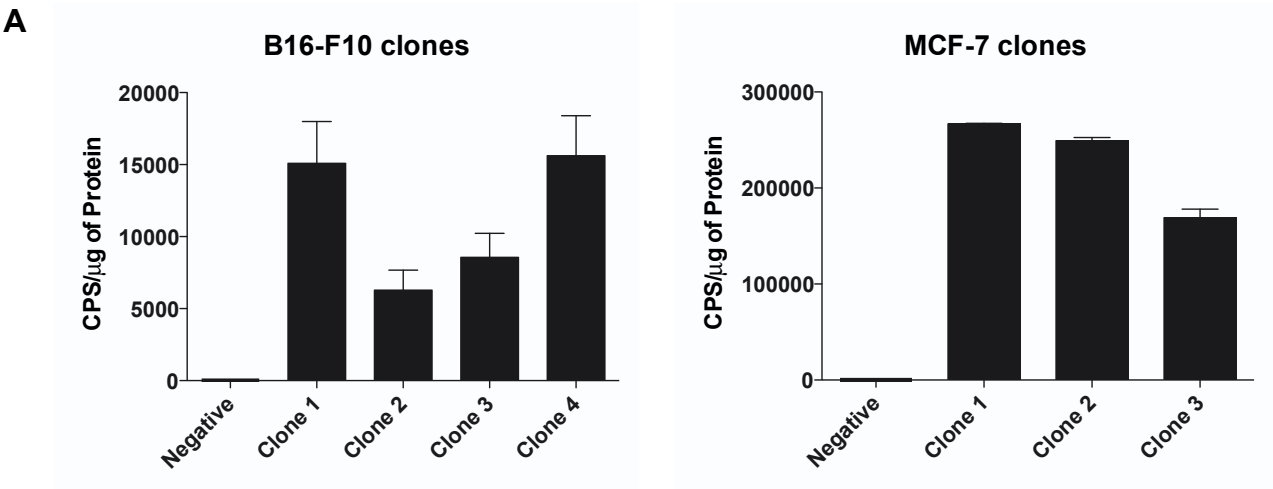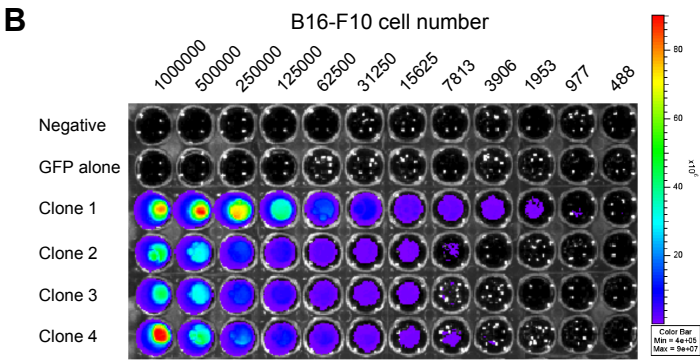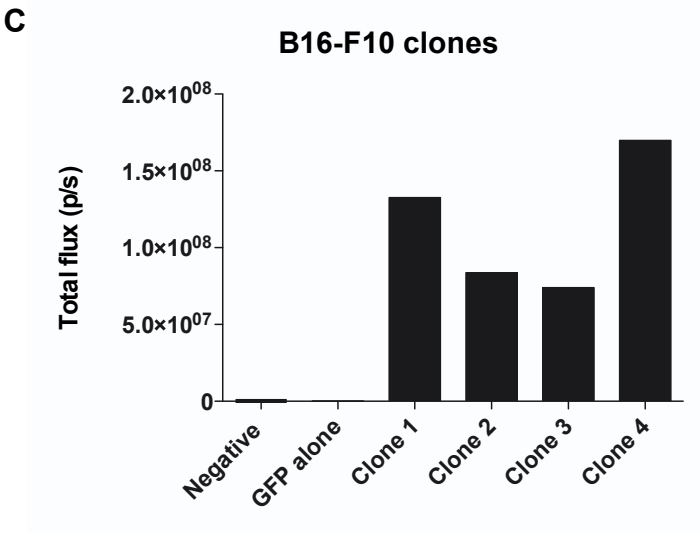

Supplement: Additional file 1 — Quantifying luciferase bioluminescence. B16-F10 and MCF-7 cells were transduced with the GFP-P2A-luc-containing lentiviral vector and GFP-positive cells were purified by FACS (> 95%). Single cell clones were isolated by limiting dilution, and expanded in vitro. B16-F10 and MCF-7 (2 × 10^6^) clonal cells were lyzed using 5X reporter lysis buffer from the Luciferase Assay System (Promega). The protein concentration was then determined using a protein assay (Micro BCA, Thermo Scientific). Cell lysate (5 μL) was mixed with 100 μL of Luciferase Assay Reagent in each well of a white 96-well plate using the microinjector on the Victor2 Wallac plate reader (Perkin Elmer). (A) Luminometry was used to measure light emissions in counts per second (CPS) over a 5 second period. Data is quantified in CPS/μg of total protein for each clone and represents 3 individual experiments performed in triplicate. (B) Luciferase bioluminescence was imaged using a Xenogen IVIS-100 by serial dilution of clones and appropriate controls (negative = untransduced, or cells transduced with GFP only). Bioluminescent images were taken following the addition of D-luciferin substrate. (C) Wells containing 1 × 10^6^ cells were quantified using Living Image software and represented as total flux measurements in photons/second. [file 1476-4598-9-299-S1.PDF]

**A**

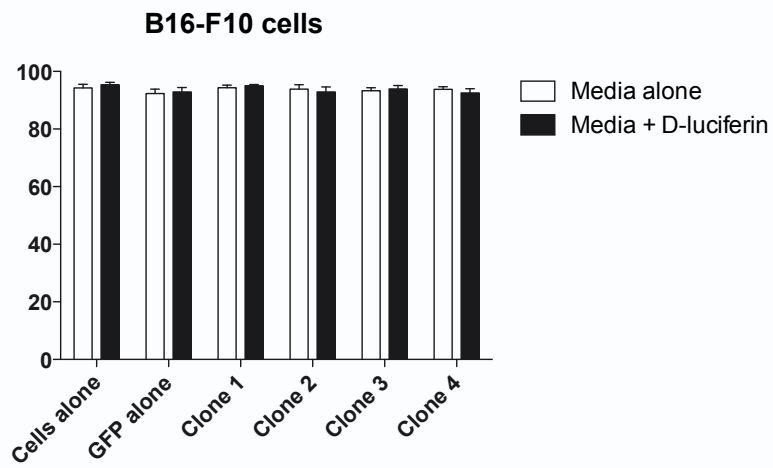

**B**

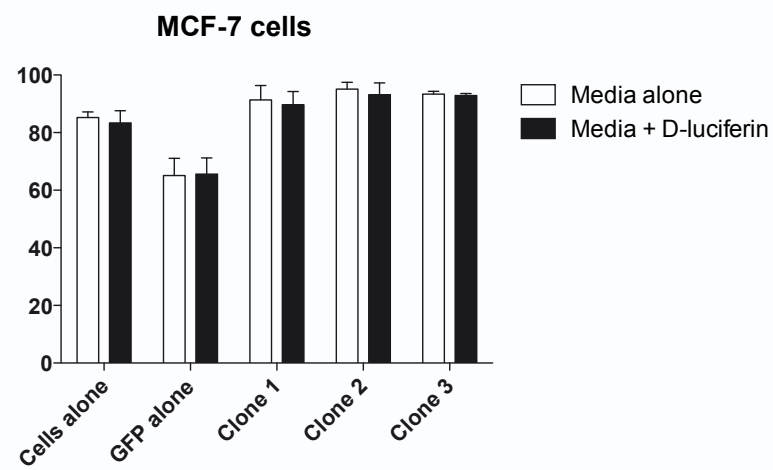

Supplement: Additional file 2 — Luciferase bioluminescence does not affect tumor cell viability in vitro. B16-F10 and MCF-7 clonal cells that stably express luciferase were seeded in 12-well plates (2 × 10^3^/well). Cells were treated with D-luciferin substrate diluted in media or media alone at 2-day intervals. Following 30 min incubation at 37°C in the dark, cells were washed once with PBS and the normal growth medium was replaced. Cell viability was measured after 8-10 days in culture using the LIVE/DEAD Fixable Dead Cell Stain Kit (Invitrogen). The violet fluorescent dye (1:500) was used to distinguish between live and dead cell populations using the BD FACSCanto flow cytometer and analyzed using Flowjo version 8.1 (Treestar). The live cell populations are represented for (A) B16-F10 cells and (B) MCF-7 cells. [file 1476-4598-9-299-S2.PDF]

**A**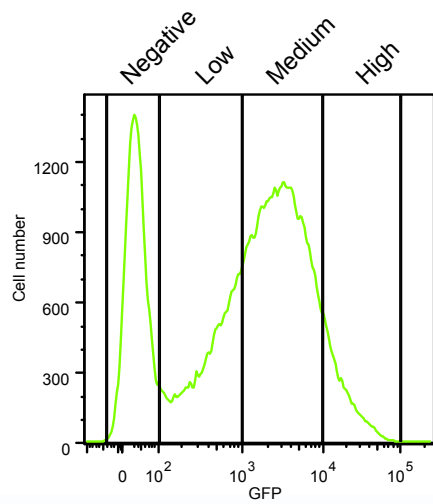**B**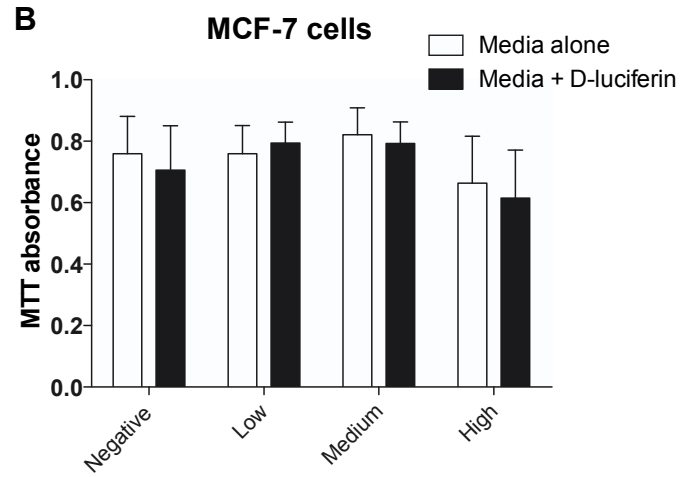**C**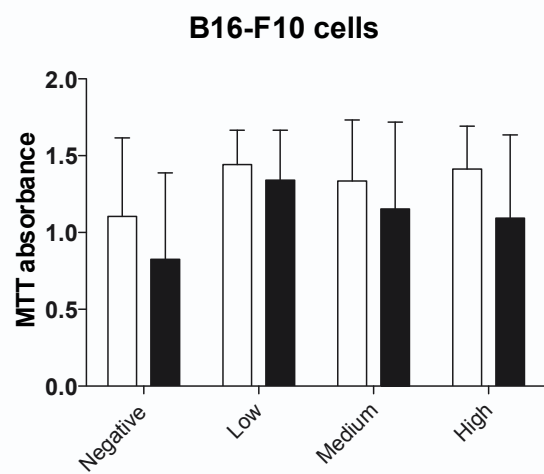**D**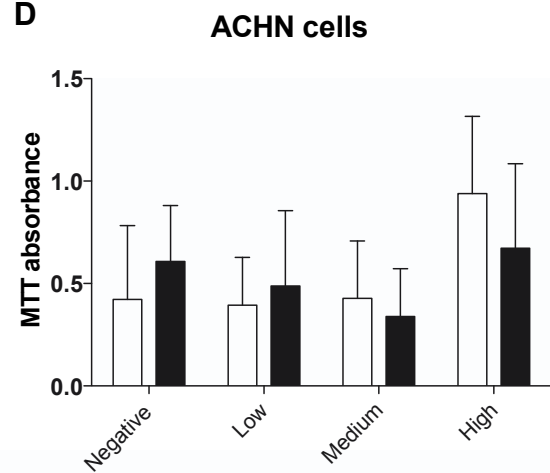**E**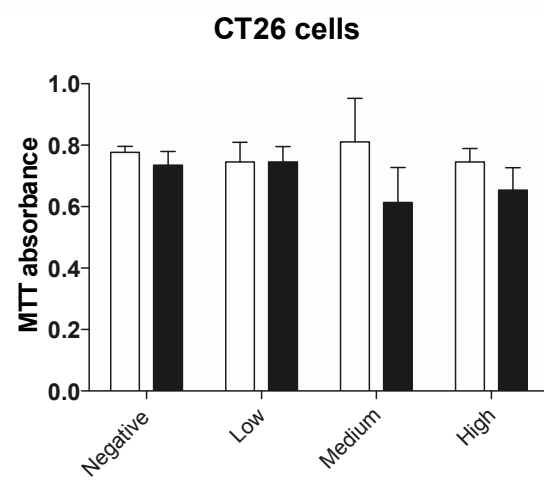**F**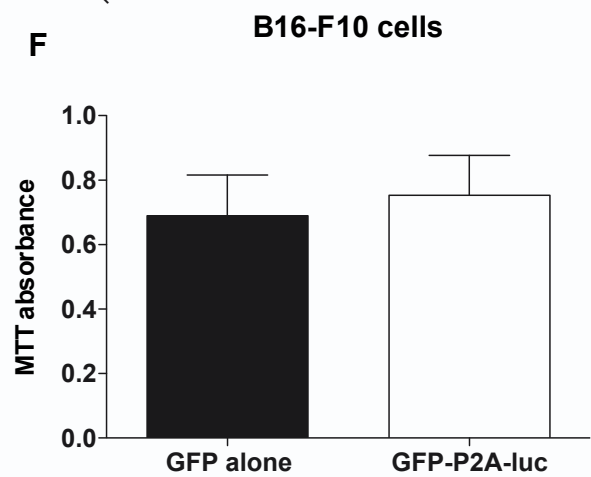

Supplement: Additional file 3 — A range of luciferase bioluminescence does not affect tumor cell viability in multiple cell types. (A) Cells were transduced with a lentiviral vector containing the GFP-P2A-luc cassette and purified into negative, low, medium or high GFP-expressing populations by FACS. Cells (400/well) were deposited directly into 96-well plates and were treated with D-luciferin substrate diluted in media or media alone at 2-day intervals. Following 30 min incubation at 37°C in the dark, cells were washed once with PBS and the normal growth medium was replaced. Cell viability was measured after 8-10 days in culture by addition of MTT substrate overnight, followed by measuring the absorbance at 572 nm for (B) MCF-7, (C) B16-F10 (D) ACHN and (E) CT26 cells. A mixed population of B16-F10 cells were transduced with lentivirus containing the GFP-P2A-luc cassette or GFP alone. (F) Identical populations of GFP expressing cells were purified by FACS and cell viability was assessed by addition of MTT substrate overnight, followed by measuring the absorbance at 572 nm. Data is shown as the mean ± s.e.m. from 3 independent experiments. [file 1476-4598-9-299-S3.PDF]

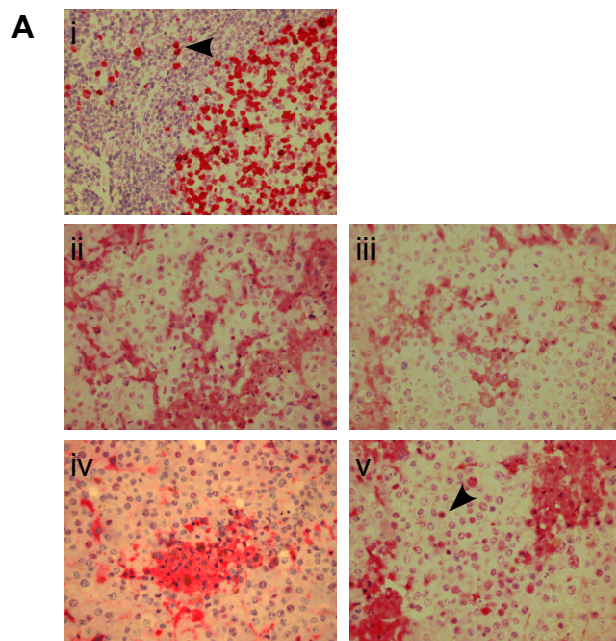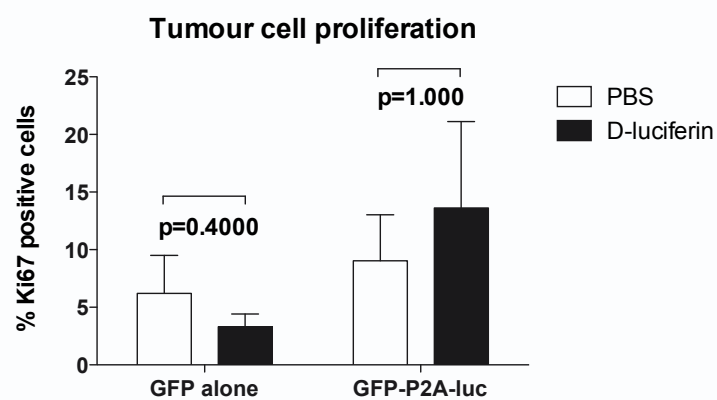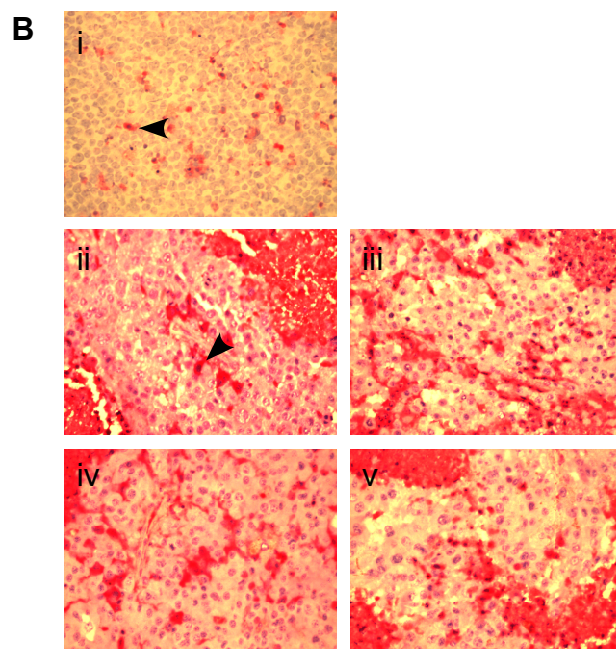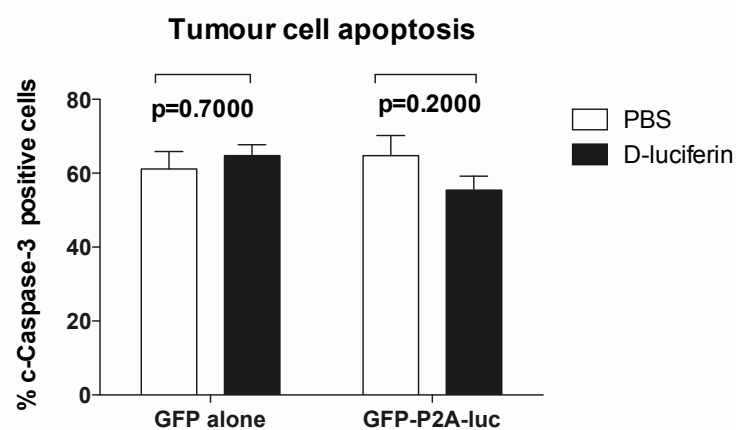

Supplement: Additional file 4 — Luciferase bioluminescence does not affect tumor cell proliferation or viability in vivo. B16-F10 tumors expressing GFP-P2A-luc or GFP alone were excised from animals and fixed in 10% neutral buffered formalin. Tumors were embedded in paraffin, cut into serial sections and mounted on lysine-coated slides. Tumor sections were stained using a Bond Max autostainer (Leica Microsystems) with an anti-Ki67 (Cat. # ORG-8772, Novocastra) or an anti-cleaved (c) caspase-3 monoclonal antibody (Cat. # 9664). A secondary antibody conjugated to alkaline phosphatase followed by chromogen staining (Fast Red) was used to distinguish between melanin and positive staining in melanocytes. Sections were counterstained with haematoxylin. (A) Ki67 staining was assessed in: i) normal tonsil tissue (positive control); ii) GFP alone tumors treated with PBS; iii) GFP alone tumors treated with D-luciferin; iv) GFP-P2A-luc tumors treated with PBS; and v) GFP-P2A-luc tumors treated with D-luciferin. Nuclear staining is indicated by arrows. (B) c-caspase-3 staining was assessed in: i) Etoposide treated Jurkat cells (positive control); ii) GFP alone tumors treated with PBS; iii) GFP alone tumors treated with D-luciferin; iv) GFP-P2A-luc tumors treated with PBS; and v) GFP-P2A-luc tumors treated with D-luciferin. Cytoplasmic staining is indicated by arrows. Slides were then scanned and whole sections were assessed for positive staining using the ACIS III system and software (Dako). Statistical significance was assessed using non-parametric (Mann-Whitney) analysis using GraphPad Prism 5.01. Data is shown as the mean ± s.e.m. from 3 tumor sections derived from one experiment. [file 1476-4598-9-299-S4.PDF]
